# Geographic Variation in Epigenetic Responses to Hypoxia in Deer Mice ( *Peromyscus maniculatus* ) Distributed Along an Elevational Gradient

**DOI:** 10.1111/mec.17752

**Published:** 2025-03-28

**Authors:** Dhriti Tandon, Shane Campbell‐Staton, Zachary Cheviron, Bridgett M. von Holdt

**Affiliations:** ^1^ Department of Ecology and Evolutionary Biology Princeton University Princeton New Jersey USA; ^2^ Division of Biological Sciences and Wildlife Biology Program University of Montana Missoula Montana USA

**Keywords:** DNA methylation, hypoxia, plasticity

## Abstract

Lowland and highland *Peromyscus maniculatus* populations display divergent, locally adapted physiological phenotypes shaped by altitudinal differences in oxygen availability. Many physiological responses to hypoxia seem to have evolved in lowland ancestors to offset episodic and localised bouts of low internal oxygen availability. However, upon chronic hypoxia exposure at high elevation, these responses can lead to physiological complications. Therefore, highland ancestry is often associated with evolved hypoxia responses, particularly traits promoting tolerance of constant hypoxia. Environmentally induced DNA methylation can dynamically alter gene expression patterns, providing a proximate basis for phenotypic plasticity. Given each population's differential reliance on plasticity for hypoxia tolerance, we hypothesised that lowland mice have a more robust epigenetic response to hypoxia exposure, driving trait plasticity, than highland mice. Using DNA methylation data of tissues from the heart's left ventricle, we show that upon hypoxia exposure, lowland mice chemically modulate the epigenetic landscape to a greater extent than highland mice, especially at key hypoxia‐relevant genes such as *Egln3*. This gene is a regulator of the gene *Epas1* that is frequently targeted for positive selection at high elevation. We find higher methylation among wild highland mice at gene *Egln3* compared to wild lowland mice, suggesting a shared epigenetic ancestral response to episodic and chronic hypoxia. These findings highlight each population's distinct reliance on molecular plasticity driven by their unique evolutionary histories.

## 1 Introduction

When species are distributed across heterogeneous environments, spatially varying selective pressures can pose distinct cost–benefit functions on traits under selection. The benefits of underlying genetic variants may supersede costs under a given environmental context, but these dynamics may be habitat dependent. In temporally or spatially varying environments; however, canalised phenotypes can be disadvantageous. Under fluctuating environmental conditions, populations often tolerate transient stressors by relying on phenotypic plasticity, which is broadly defined as the ability of one genotype to produce multiple phenotypes in response to environmental conditions (Boutin and Lane 2014; Hetem et al. 2014; Parmesan and Yohe 2003; West‐Eberhard 2003). Plasticity is often, though not always, an adaptive mechanism for tolerating transient stressors while minimising costs associated with constitutive trait expression (Ghalambor et al. 2007; Walther et al. 2002). For example, seasonal phenotypes, such as diapause in insects (Pegoraro et al. 2016), the activation of the reproductive system in mammals (He et al. 2021) or migration timing in birds (Saino et al. 2017, 2019), are both plastic and associated with genetic variation, epigenetic modifications and environmental factors (Fishman and Tauber 2024).

Epigenetic modifications provide a proximate mechanism underlying phenotypic plasticity with ecological relevance (Crossman et al. 2021; Laubach et al. 2019; Pinho et al. 2022). Changes in the reversible state of adding a methyl group (—CH_3_) to the 5' carbon of a cytosine (Razin and Riggs 1980) that alter patterns of gene expression in response to environmental stimuli are among the best characterised of these mechanisms (e.g., glycolysis regulation associated with baboon diets based on natural forage or human food scraps, Lea et al. 2016). Cytosine methylation proximal (*cis*‐) to a transcription start site can reduce gene expression in most cellular contexts by blocking DNA binding sites for *trans*‐acting transcription factors (Smith and Meissner 2013). Conversely, cytosine methylation at specific sites within gene bodies may be associated with modified stable mRNA levels and alternative splicing (Lorincz et al. 2004; Maor et al. 2015).

Due to its rapid and reversible nature, DNA methylation also provides a proximate explanation for evolutionary persistence in the face of environmental change through adaptive phenotypic plasticity (Angers et al. 2010; Childebayeva et al. 2021; Law and Holland 2019; Simpson 1953). Such epigenetic plasticity may be especially beneficial when organisms live in fluctuating environments, where temporary adjustments have selective advantages over canalised modifications (Herman and Sultan 2016). Plasticity, though, can be costly due to organismal investment into epigenetic machinery and sensory systems (Herman and Sultan 2016), as well as antagonistic pleiotropic outcomes of behavioural and physiological modifications (Morris and Rogers 2013; Schneider and Meyer 2017). Moreover, plastic responses that evolved in a specific environmental context (i.e., ancestral plasticity) can also produce maladaptive outcomes when they are induced in novel contexts, for example, habitat conversions or postcolonisation of new habitats (Morris and Rogers 2013; Schneider and Meyer 2017). When the plasticity costs exceed its benefits, we expect evolutionary modification of ancestral plastic responses (i.e., genetic accommodation, West‐Eberhard 2003) or potentially the loss of plasticity entirely (i.e., genetic assimilation, Waddington 1942, 1959; West‐Eberhard 2003). If transient environmental stressors become chronic and permanent, an evolved loss of plasticity is expected where genetic variants are favoured that blunt ancestral plasticity and promote genetically determined fixed trait expression (Stager et al. 2024; Velotta and Cheviron 2018).

The colonisation of high‐elevation habitats is a good example of the transition from a transient to chronic environmental stressor. Most vertebrate hypoxia responses likely evolved in lowland ancestors to offset transient or localised tissue‐level hypoxia (Storz et al. 2010). Lowland organisms routinely experience hypoxia at multiple levels of biological organisation and at different spatial and temporal scales through a wide variety of means including malnutrition (i.e., anaemia), injury or exercise. When colonising high elevation from lowland habitats, organisms are faced with chronic reductions in oxygen availability. Consequently, when physiological systems that evolved to offset transient and localised hypoxia are subjected to chronic and global hypoxia, they can produce misdirected outcomes that suppress aerobic performance and reduce fitness (Storz et al. 2010). One prominent example is the hypoxic pulmonary vasoconstrictive response. In lowland vertebrates, pulmonary blood vessels constrict in response to regional, tissue‐level hypoxia. This response is beneficial at low elevations because it helps to direct blood to well‐oxygenated regions of the lung, which matches blood flow to regional variation in ventilation across the lung and improves the efficiency of gas exchange. However, this response is counterproductive when it is induced to offset the global, rather than regional, hypoxia experienced at high elevation. This vasoconstriction occurs across the entire lung and results in thickening of the pulmonary vessels, which reduces their distensibility and leads to increased pulmonary arterial blood pressure (Sylvester et al. 2012; Shimoda and Laurie 2014). This induced lowland response to hypoxia reduces the efficiency of gas exchange and can lead to life‐threatening conditions (Sylvester et al. 2012; Storz and Scott 2021). Accordingly, we expect an evolved loss of plasticity in derived highland taxa (Storz et al. 2010; Storz and Scott 2021).

An example of a derived loss of plasticity can be found in highland populations of the North American deer mouse ( *Peromyscus maniculatus* ) (Storz and Scott 2021; Velotta et al. 2018). Deer mice are among the most widespread mammals in the western hemisphere, continuously distributed across a large elevational range from geographic regions below sea level to the summits of the highest peaks in North America (Hock 1964). Highland mice have evolved several physiological adaptations that span the entirety of the oxygen transport cascade and improve aerobic performance under hypoxia (reviewed in Storz et al. 2019). Evolved physiological changes that promote increased fitness in highland deer mice include modifications of the hypoxic ventilatory response (Ivy and Scott 2017), greater pulmonary O_2_ diffusion (Tate et al. 2017), improved cardiovascular function (Schweizer et al. 2019; Tate et al. 2017, 2020), greater blood oxygen affinity (Chappell and Snyder 1984; Natarajan et al. 2013; Storz et al. 2009, 2010), greater skeletal muscle oxidative capacity and capillarity (Lau et al. 2017) and improved mitochondrial function (Dawson et al. 2018; Mahalingam et al. 2017; Scott et al. 2018). Complementing these adaptations in oxygen supply and consumption pathways are modifications of fuel supply and oxidation pathways (Cheviron et al. 2012, 2014; Lyons et al. 2021; Lyons and McClelland 2022; Velotta et al. 2016, 2018). In combination, the adaptive modification of these integrated physiological processes allows for adequate supply of oxygen and metabolic fuels to thermogenic organs that power exceptionally high rates of aerobic thermogenesis in highland deer mice, a trait that is known to enhance survival at high elevation in this species (Hayes and O'Connor 1999).

Moreover, many of these performance‐associated traits are constitutively expressed in highlanders but plastic in lowlanders, suggesting an evolved blunting of plastic responses to hypoxia in highland mice (Storz et al. 2019; Storz and Scott 2021; Velotta and Cheviron 2018). This pattern is especially evident in that highland deer mice have evolved a blunted pulmonary vasoconstrictive response to hypoxia, while lowlanders exhibit a robust response and a dramatic increase in hypertrophy of the right ventricle, an outcome of pulmonary hypertension and a symptom of chronic mountain disease (Velotta et al. 2018; West et al. 2021). Highland deer mice also exhibit reduced ventilatory (Ivy and Scott 2018; Ivy et al. 2022) and erythropoietic responses (Mahalingam et al. 2017) to hypoxia. Together, these observations suggest evolutionary divergence in plastic responses to hypoxia in populations of deer mice that are adapted to different elevational environments.

The mechanistic and genetic bases of population differences in hypoxia responses in deer mice have, at least in part, been associated with genetic variants in key hypoxia signalling pathways. For example, the endothelial PAS domain‐containing protein 1 (*Epas1*, encoding the hypoxia‐inducible factor 2α, HIF‐2α), encodes the oxygen‐sensitive subunit of the dimeric transcription factor HIF2, which regulates several systemic responses to hypoxia (Befani and Liakos 2018; Hodson et al. 2016; Petousi et al. 2014; Schweizer et al. 2019). A derived amino acid substitution is found at high frequency (> 80%) in highland deer mice (*Epas1* ^Thr^755^Met^), and this allele is associated with population differences in ventilatory and cardiac responses to hypoxia and altered expression of genes implicated in catecholamine biosynthesis and secretion (Ivy et al. 2022; Schweizer et al. 2019). This mutation results in a loss of function, impairing HIF‐2α binding to its coactivator, the CREB‐binding protein (Song et al. 2021). Such a loss of function may contribute to the blunting of some highlander responses to hypoxia (which diverged from their lowland ancestors approximately 217,000 generations ago, Schweizer et al. 2019), but the relative contributions of other variants and other mechanisms such as epigenetic modifications are poorly understood. While not directly tested, the benefits of this mutation may be outweighed by costs in lowland mice due to a loss in the multifunctional HIF‐2α activity (Kirschner et al. 2022; Suzuki et al. 2016). We, therefore, suspect that a population's reliance on specific molecular pathways to achieve hypoxia tolerance is determined by their geographical context, either supporting plastically derived or constitutively expressed traits (Storz et al. 2019; Storz and Scott 2021; Velotta and Cheviron 2018). Specifically, since permanent genetic variants supporting hypoxia tolerance may be maladaptive in lowland conditions, populations living within these habitats could rely on reversible molecular switches to drive plastic phenotypic responses to transient stressors in a fluctuating or rapidly changing environment.

We hypothesised that deer mice derived from lowland and highland populations have distinct methylation responses to experimental hypoxia that accumulate in biological pathways and show signatures of elevation‐dependent selection among lowland and highland mice (Schweizer et al. 2019, 2021) and are differentially regulated in response to hypoxia. We further expected a greater epigenetic response to hypoxia in lowland mice compared to highlanders, which would exhibit the utility of plasticity in lowlanders rather than irreversible sequence‐level polymorphism to tolerate this stressor. Briefly, to test our hypotheses, we conducted a series of experimental hypoxia exposures on offspring of wild‐derived mice that have evolved either at high (4350 m above sea level) or low (430 m) elevations, or to wild mice living in their natural environments. After acclimating, we assessed cytosine methylation of cardiac tissue, which undergoes plastic physiological responses to hypoxia (Williams et al. 2022) and identified differences in methylation in response to hypoxic conditions for each population.

## 2 Materials and Methods

### 2.1 Sample Collection

We exposed 28 laboratory‐born and reared highland and lowland deer mice to experimental hypobaric hypoxia to assess population‐specific methylation responses to hypoxia exposure (Supporting Information). Lowland mice were derived from wild‐caught adults collected in eastern Nebraska (Lincoln; Lancaster County, Nebraska, USA; 40°52′ 12′′ N, 96°48′ 20.3′′ W; 430 m above sea level; standard pO_2_ = 20.2 kPa) and highland mice from the summit of Mt. Blue Sky (formerly Mt. Evans) in the Rocky Mountains (Clear Creek County, Colorado, USA; 39°35′ 18′′ N, 105°38′ 38′′ W; 4350 m above sea level; standard pO_2_ = 12.8 kPa). We acclimated lab‐born, age‐matched adult lowland and highland mice in mixed‐sex groups to (1) normobaria (n_Lowland_ = 7; n_Highland_ = 10) in standard normoxic holding conditions, or (2) hypobaric (lower than standard oxygen levels) hypoxia (n_Lowland_ = 4; n_Highland_ = 7) [barometric pressure of 60 kPa; equivalent to the elevation on the summit of Mt. Blue Sky (~4300 m), where the highland mice are derived from] in hypobaric chambers that have been described previously (Lui et al. 2015; McClelland et al. 1998; Velotta et al. 2020) (Table S1). Following acclimation for 6 weeks, we euthanised individuals using an overdose of isoflurane followed by cervical dislocation. We removed whole hearts and dissected and isolated left ventricles, which were then flash frozen on liquid nitrogen. Left ventricle tissue was chosen due to its ability to mediate cardiac output and other plastic physiological responses to hypoxia (Williams et al. 2022). To characterise methylation patterns in wild mice living in native environments, we also collected left ventricle tissue from wild mice captured either on the summit of Mt. Blue Sky (*n* = 5) or Lincoln (*n* = 6). Mice were euthanised at the site of capture and tissues were frozen on liquid nitrogen.

### 2.2 DNA Preparation and Genomic Library Construction

We collected DNA methylation data using reduced representation bisulphite sequencing (RRBS), which enriches the genomic libraries for regions with high CpG content and are most likely to contain biologically relevant regulatory sites, promoters and repetitive regions (Boyle et al. 2012; Meissner et al. 2005). We extracted genomic DNA from left ventricle tissue following Qiagen DNeasy's protocol (Qiagen). Briefly, we digested high molecular weight genomic DNA with the *Msp1* restriction enzyme with subsequent preparation following the NEBNext sample preparation kit protocol (New England Biolabs). We spiked in 1 ng of enterobacteria phage λ DNA to each library as a nonmethylated internal control for estimating the efficiency of bisulphite (BS) conversion downstream (Lea et al. 2016). We used AMPure beads for DNA purification and to retain fragments between 100 and 400 bp in size. The resulting fragments were treated with BS to convert unmethylated cytosines following the low DNA input (1 ng–2 μg) protocol in the Qiagen EpiTect Fast Bisulfite Conversion kit (Qiagen). We conducted low amplification of converted DNA with 12 cycles of PCR to enrich for adapter‐ligated fragments with MyTaq Mix (Bioline Inc.), during which each sample was also barcoded with unique sequence tags that permitted the pooling of final genomic libraries. We collected single‐end (1 × 100 nt) SP sequencing on an Illumina Novaseq6000 in Princeton University's Lewis Sigler Genomics Core Facility.

### 2.3 Bioinformatic Processing

We prepared the reference *Peromyscus maniculatus* genome (Pman_2.0) for alignment of BS‐converted sequence reads using *bowtie2* in *BS‐Seeker2 v2.0.10* and bounded from 50 to 500 bp with no more than four mismatches (Chen et al. 2010; Guo et al. 2013; Langmead 2010; Langmead and Salzberg 2012). We demultiplexed sequence pools by retaining reads that had a perfect sequence match to the expected barcode sequence tags with an in‐house Python script. We removed low‐quality reads (Q < 20), clipped adapter sequences and discarded reads that are < 20 bp in length using *cutadapt 1.8.1* (Martin 2011). We mapped trimmed reads to the reference using the *bs‐seeker_2‐align.py* script in *BS‐Seeker2* (flags included: *‐r ‐L* 50 *‐U* 500). We used default settings for the *bs_seeker2‐call‐methylation.py* script and the *‐x* flag to remove reads not fully converted by the bisulphite treatment. We estimated the conversion efficiency by mapping each genomic library to the 48,502 bp phage lambda linear genome (NC_001416.1) and assessed methylation frequency (MF), which is the proportion of methylated cytosines to the total sequencing depth at that nucleotide position (Chen et al. 2010), of the converted λ DNA as [*1‐average MF across the phage* λ *genome*].

We then constructed a single dataset with all individuals using the R v4.2.1 (R Core Team 2021) program *methylKit v1.24.0* (Akalin et al. 2012), using the *methRead* function (*context =* ‘CpG’, *mincov =* 10) to normalise read depth into a per‐cytosine MF with a minimum of 10‐fold sequencing coverage. We employed a principal component analysis (PCA) using the *PCASamples* function to identify potential outliers for exclusion. We then constructed a dataset unique to each population that also included a maximum read threshold of 95% percentile. Given our goal to conduct a comparative analysis of differentially methylated sites from both datasets, we equalised the significance thresholding across the two datasets such that we apply FDR corrections to the same number of sites in both datasets (Supporting Information). Therefore, we retained only the sites that were present in both datasets. We restricted our analysis to the CpG motifs given their established role in vertebrate methylation and excluded methylation marks in the CHH and CHG motifs (Suzuki and Bird 2008).

### 2.4 Population‐Specific Differentially Methylated Sites

As methylation at most mammalian CpG sites is bimodal (Cedar and Bergman 2012), this violates normality assumptions for a two‐way *ANOVA*, even after data transformations. We used logistical regression‐based differential methylation analyses on *methylkit* for each population separately to identify population‐specific differentially methylated cytosines in hypoxic versus normoxia conditions. After filtering sites with low coverage, we applied the *calculateDiffMeth* function with the experimental oxygen treatment of hypoxia or normoxia as the explanatory variable, with sex, sequencing pool and the parental breeding pair ID as proxy variables for relatedness as covariates. We used the default threshold of statistical significance to identify differentially methylated sites (DMSs) as cytosines with methylation differences (*methDiff*) greater than 25% and a false discovery rate (FDR) corrected threshold *p <* 0.05, re‐estimated using the *p.adjust* on R *v4.2.1* along common sites in both analyses. We statistically evaluated differences in the proportion of DMS with respect to the total sites tested by using a Pearson Chi‐square test of independence with a 2 × 2 contingency table (R function *chisq.test*) that contained the number of sites identified as DMS and the number of remainder sites (total sites *minus* DMS) among highland and lowland individuals. To assess if DMSs were physically clustered and potentially formed a differentially methylated region (DMR), we highlighted cytosines that were located ± 1 kb of the DMS and approached significance when the region itself displayed methylation signatures in the same direction as the reported DMS. We were unable to estimate DMRs using standard bioinformatic pipelines due to the sparsity of the reduced representation method.

We annotated the genomic features associated with each of the DMSs to gain insights into the putative functional relevance of the differential methylation using the *bedtools v2.30.0* (Quinlan and Hall 2010) *intersect* functionality against the reference genome GFT annotation v105 file. We considered a site to be associated with a given gene if it was within the gene boundary or < 1 kb from the gene boundary as defined in Ensembl. We included the 1 kb flanking the gene boundary as DNA methylation can impact gene expression through its activity in regulatory regions located in *cis*‐ conformations outside gene boundaries, in addition to its molecular impacts in coding regions (Dai et al. 2020; Tycko 2010). After assigning gene(s) associated with each DMS, we further annotated each DMS as hypoxia‐relevant or ‘other’ by manually cross‐referencing its associated gene ID to a list of hypoxia‐relevant genes (Schweizer et al. 2019). We repeated the Pearson Chi‐square test of independence for the number of sites identified as ‘DMS and hypoxia‐relevant’ and the number of all remaining sites tested among highland and lowland individuals. To test whether the proportion of called DMS identified as hypoxia‐relevant were different between highland and lowland individuals, we additionally repeated the Chi‐square test for the number of sites identified as ‘DMS and hypoxia‐relevant’ and ‘DMS and “other”’. Following from our expectations for differences in *Epas1*‐related regulation among highland and lowland individuals, we obtained gene names of the regulators of *Mus musculus* *Epas1* from *STRING* (Szklarczyk et al. 2015) v11.5 database and noted whether *Epas1* or any of its regulators harboured DMS in each population.

### 2.5 Comparing DNA Methylation to the Wild

To contextualise lab‐based findings to conditions in the wild, we repeated the differential methylation analysis in wild mice with population (lowland vs. highland) as the explanatory variable and included sex and sequencing pool as covariates. For sites that were statistically significant (−25 < methDiff > 25 and *q* < 0.05), we used *bedtools v2.30.0 intersect* to find common differentially methylated sites between (1) ‘highland versus lowland’ analyses from wild mice and ‘hypoxia versus normoxia’ analyses from lab‐derived lowland mice; and (2) ‘highland versus lowland’ analyses from wild mice and ‘hypoxia versus normoxia’ analyses from lab‐derived highland mice. Using *bedtools v2.30.0* intersect, we also explored whether the differentially methylated sites that are representative of DNA methylation differences in the wild are found within hypoxia‐relevant genes. Finally, we estimated average methylation frequencies for mice exposed to normoxic and hypoxic conditions for each population. To qualitatively assess if a treatment deviated from a 1:1 relationship of equal methylation averages in each treatment, we generated X‐Y scatterplots of normoxia and hypoxia averages. A larger deviation from this relationship would indicate a greater magnitude of change and a more robust methylation response between normoxic and hypoxic conditions.

### 2.6 Gene Ontology Enrichment Analyses and Functional Consequences

We assessed differences in functional enrichment of epigenetic responses using a gene ontology (GO) enrichment analysis for the gene(s) associated with each DMS with *g:Profiler* (Raudvere et al. 2019) using the *Peromyscus maniculatus* as the reference genome. For the background comparison, we included all genes that were sequenced in the RRBS genomic libraries and passed quality filtering. We used the default significance threshold = ‘g:SCS’, user threshold = 0.05. To decipher population‐specific differences in associated functional pathways, we completed the GO enrichment analyses separately for genes containing DMS in each population.

## 3 Results

We sequenced 28 deer mice sampled from lowland (*n* = 11) and highland (*n* = 17) habitats and obtained an average of 25,897,716 reads that mapped uniquely per sample that covered 4.3 million bases (Tables S1, S2). Mappability levels were acceptable (average = 63.7%) and we found that cytosines were enriched in the CG motif (per cent of CG = 24.9%, CHG = 2.69%, CHH = 1.79%) with high conversion proportions (Table S2). We found that the first two PC axes explained 21.9% and 4.83% of the variance in DNA methylation across samples, respectively, and identified a single sample as an outlier that was removed from all downstream analyses (Figure S1; Table S1). We constructed the respective datasets (lowlanders n_hypoxia_ = 4, n_normoxia_ = 7; highlanders n_hypoxia_ = 7, n_normoxia_ = 9) where we retained 1,568,859 cytosines for further analyses (Table S1). Thereafter, we surveyed population‐specific epigenetic changes in response to hypoxia by identifying differentially methylated cytosines as an effect of acclimation treatment (normoxia vs. hypoxia) among the lowland and highland populations separately.

### 3.1 Unique DMSs in Lowland Mice

**FIGURE 1:**
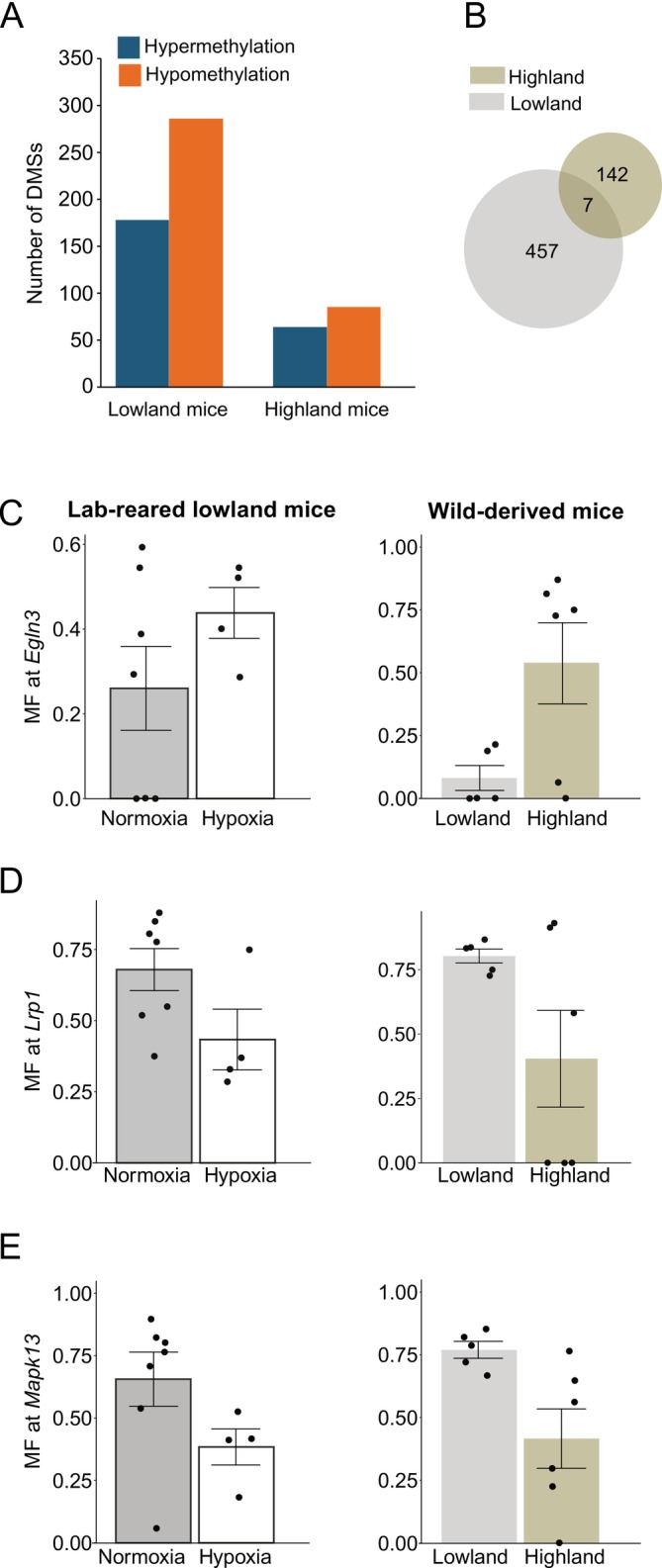
Summary of population‐specific methylation responses. (**A**) Bar graph denoting the number of hypermethylated and hypomethylated differentially methylated sites (DMSs) for lowland and highland mice; (**B**) Venn diagram depicting a number of unique DMS to lowland and highland mice. Intersection depicts common DMS among lowland and highland mice. Average (and standard error) methylation at DMS located within (**C**) *Egln3* (chr14:16866983), (**D**) *Lrp1* (chr18:44654244) and (**E**) *Mapk13* (chr21:5927643) among lab‐reared lowland mice and wild‐derived mice. Abbreviation: MF, methylation frequency.

**FIGURE 2:**
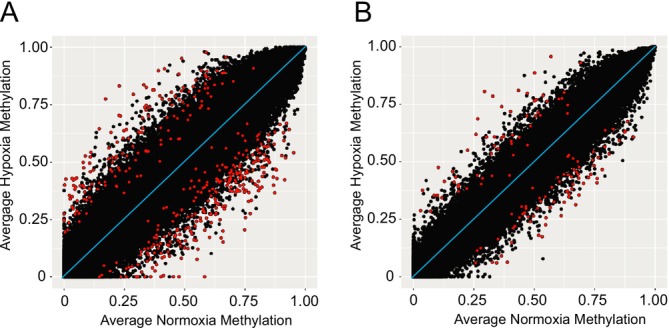
Global methylation response to hypoxia in lowland and highland mice. The relationship of average methylation frequencies in hypoxia and normoxia treatments across all retained cytosines for (**A**) lowland and (**B**) highland lab‐reared mice. Red points indicate differentially methylated sites; the diagonal line represents no methylation differences between treatments.

We found that significantly more sites were differentially methylated in response to hypoxia in lowland mice (*n* = 464) compared to highland mice (*n* = 149) (χ^2^ = 160.87, *p* < 2.20 × 10^−16^) (Figure 1A) (Supplementary File 1). Of the 464 total DMSs in lowlanders, 178 of the cytosines were hyper‐methylated (methDiff > 25, *p*_*adj*_ < 0.05) and 286 were hypo‐methylated (methDiff < −25, *p*_*adj*_ < 0.05) in response to hypoxic conditions. Highland mice had 64 hyper‐methylated sites (methDiff > 25, *p*_*adj*_ < 0.05) and 85 hypo‐methylated sites (methDiff < −25, *p*_*adj*_ < 0.05) in response to hypoxic conditions. We found that lowland mice tended to have significantly more DMS located in hypoxia‐relevant genes (*n* = 38) than highland mice (*n* = 10) (χ^2^ = 15.2, *p* = 9.73 × 10^−5^). However, of the total DMS, the number of DMS at hypoxia‐relevant genes was not statistically different among lowland and highland mice (DMS_Hypoxia_/DMS_Total_ lowland = 0.082 and highland = 0.067 respectively; χ^2^ = 0.16; *p* = 0.68). Among the genes that bear signatures of positive selection in the highland population of deer mice (Schweizer et al. 2019), lowland mice had more DMS (*n* = 6) compared to highland mice (*n* = 1); however, this difference was not statistically significant (χ^2^ = 0.10; *p* = 0.75). We only found seven overlapping DMS between highlanders and lowlanders, of which two had the same direction of methylation change (Figure 1B; Table S3). Five of the seven common DMS are associated with genes, of which one site was within the hypoxia‐relevant TNF Superfamily Member 13 (*Tnfsf13*) gene: hyper‐methylated in lowland mice and hypo‐methylated in highland mice (Table S3). Lowland mice exposed to hypoxic conditions carried a single hyper‐methylated DMS located within the intron of the gene Egl‐9 family hypoxia‐inducible factor 3 (*Egln3*, chr14:16866983, methDiff = 26.9, *p*_*adj*_ = 0.0432), which is a regulator of endothelial PAS domain protein 1 (*Epas1*) gene (Figure 1C). We found that cytosines around the chr14:16866983 DMS (+/− 1 kb) and approaching significance (i.e., *p* < 0.05; *p*_*adj*_ > 0.05) also showed evidence for hypermethylation in hypoxia‐exposed lowland mice (Figure S3A). While the chr14:16866983 *Egln3* locus also had increased methylation under hypoxia in highland mice, this difference was not significant after multiple test corrections (methDiff = 24.3, *p* = 0.031; *p*_*adj*_ = 1) (Figure S2A). We did not find any DMS within or upstream of *Epas1* or its regulators for highland mice. We also found that lowlanders have a more pronounced genome‐wide methylation response to hypoxia than highlanders, as evidenced by greater deviation from the line of equality, which represents a higher magnitude of change in methylation between normoxic and hypoxic treatments (Figure 2). We did not find any enriched GO terms upon using a custom background list of genes containing all retained cytosines in our analysis.

### 3.2 A Subset of Differentially Methylated Sites Under Experimental Hypoxia Overlapped With Methylation Differences in the Wild

To assess whether DNA methylation differences driven by hypoxia represent naturally occurring epigenetic differences between highland and lowland mice in the wild, we also collected and analysed data from 11 wild‐caught mice (highland *n* = 6, lowland *n* = 5). For these wild mice, we obtained an average of 23,614,779 reads that mapped uniquely per sample and covered 2.3 million bases (Table S2). Mappability levels and CG enrichment were similar to data derived from the lab‐reared mice (Table S2). We found 3622 DMSs among wild lowland and highland mice (Supporting Information), 30 of which were differentially methylated among lowland lab‐reared mice exposed to normoxic versus hypoxic treatments, and one of these DMSs was located within the intron of *Elgn3* (chr14:16866983) with higher methylation in low oxygen conditions (i.e., lowland lab‐reared hypoxic mice and wild highland mice (Wild methDiff_[low vs. high]_ = 45.8; Lowland/Lab‐reared methDiff_[normoxia vs. hypoxia]_ = 26.9) (Figure 1C). Analogous to lowland mice, a suggestive DMR that contained the *Egln3* DMS also showed evidence of hypermethylation in wild highland mice (Figure S3B). Other hypoxia relevant genes that harboured a DMS in the same direction of methylation change between ‘wild lowland versus highland analyses’ and ‘lowland derived lab‐reared normoxia versus hypoxia analyses’ include (1) a hypomethylated DMS within the intron/splice region of LDL receptor–related protein 1 (*Lrp1*, chr18:44654244; Wild methDiff_[low vs. high]_ = −41.0; Lowland/Lab‐reared methDiff_[normoxia vs. hypoxia]_ = −27.4) (Figure 1D), a modulator of vascular homeostasis (He et al. 2021); and (2) a hypomethylated DMS within the exon of mitogen‐activated protein kinase 13 (*Mapk13*, chr21:5927643; Wild methDiff_[low vs. high]_ = −34.8; Lowland/Lab‐reared methDiff_[normoxia vs. hypoxia]_ = −33.7; Figure 1E), a regulator of the vascular endothelial growth factor (Duyndam et al. 2003). We did not find consistent evidence for hypomethylation (methDiff < 0; *p* < 0.05) for highland wild and hypoxia‐exposed lowland mice among cytosines located near *Lrp1* (Figure S3C,D). While a potential DMR that contained the *Mapk13* DMS also showed evidence for hypomethylation in wild highland mice (Figure S3F), only 50% of the cytosines in the DMR showed evidence for hypomethylation (Figure S3E). We also found a hypermethylated DMS within the exon of matrix metalloproteinase 17 (*Mmp17*, chr23:20786806) (Huang et al. 2009), in wild highland mice (methDiff_[low vs. high]_ = 45.7); however, low‐oxygen related hypomethylation of this site in Lowland/Lab‐reared mice (methDiff_[normoxia vs. hypoxia]_ = −48.3). We additionally identified 20 sites that were differentially methylated among lab‐reared highland mice exposed to hypoxic versus normoxic conditions, and wild mice derived from lowland versus highland habitats. None of these were within hypoxia‐relevant genes. We found a single DMS downstream of the RAS guanyl‐releasing protein 4 (*Rasgrp4*) gene (chr1:39905168, *Pman_2.0*) that was common to all three analyses and hypomethylated in hypoxia‐treated lowland‐derived lab mice (normoxia vs. hypoxia methDiff = −32.5), hypermethylated in hypoxia‐treated highland‐derived lab mice (methDiff = 47.9) and hypermethylated in wild highland mice (vs. lowland mice, methDiff = 31.0). While not flagged as a hypoxia‐relevant gene in mice (Schweizer et al. 2019), this gene encodes a member of the RAS family of proteins, which have been shown to induce signalling pathways antagonising HIF‐1 expression in *Caenorhabditis elegans’* vulval precursor cells (Maxeiner et al. 2019). Overall, we did not find consistent evidence for concordant methylation changes (*p* < 0.05) in cytosines ± 1 kb of this DMS in all three analyses (Figure S4).

## 4 Discussion

### 4.1 Distinct Reliance on Molecular and Phenotypic Plasticity

Distinct environments present different costs and benefits on phenotypic outcomes. As a result, genetically determined adaptations to a trait that may be beneficial in one population could be deleterious in another. In environments characterised by transient exposure to stressors, reversible molecular switches may be favoured over genetic mutations that encode fixed trait expression (Maxeiner et al. 2019). Our study revealed population‐specific epigenetic responses to hypoxic conditions. Specifically, we found that lowland mice generally exhibited a more pronounced methylation response (i.e., more DMS and greater magnitude of methylation difference) to hypoxia with very little overlap among the differentially methylated sites between highlanders and lowlanders. This pattern of methylation plasticity mirrors that in other higher level phenotypic traits such that lowland mice have more pronounced plastic responses to hypoxia across a wide range of physiological pathways (Lui et al. 2015; Storz et al. 2019). These observations suggest evolutionary blunting of ancestral phenotypic plasticity (i.e., genetic assimilation), whereby physiological and regulatory systems that evolved to offset transient and localised low oxygen exposure in ancestral lowland mice were modified following generations of exposure to chronic low oxygen conditions in derived highland mice. Such conditions may favour the permanent expression of phenotypic traits that improve oxygen transport and consumption, as well as blunted physiological responses to hypoxia (Ivy and Scott 2018; Storz et al. 2019; Storz and Scott 2021; Velotta et al. 2018).

A derived loss of physiological plasticity is common in high‐altitude specialists, including highland deer mice (Storz et al. 2010; Storz and Scott 2021). Prominent examples include the blunting of the pulmonary vasoconstriction response and the excessive erythropoiesis that characterise lowlander responses to hypoxia (Storz et al. 2010; Storz and Scott 2021). Highland deer mice have evolved a blunted pulmonary vasoconstrictive and erythropoietic response to temporal and global chronic hypoxia, while lowlanders exhibit a robust response that includes a dramatic increase in hypertrophy of the right ventricle and in haematocrit (Velotta et al. 2018; West et al. 2021). Our results here suggest that this pattern of evolutionary blunting of hypoxia responses extends to epigenetic modifications that may influence gene regulation as well.

### 4.2 Implications of DNA Methylation‐Induced Plasticity

Lowland mice exhibited a more pronounced methylation response in a subset of genes that are known to be involved in physiological responses to hypoxia. In addition, we found that hypoxia exposure in lowland mice recapitulates some epigenetic differences between lowland and highland mice in the wild, as evidenced by the same direction of methylation change across three hypoxia‐relevant genes. One example is gene *Egln3* that encodes the protein PHD‐3 that regulates the oxygen‐sensitive transcription factor HIF2, which, in turn, regulates several physiological responses to hypoxia and is known to be a repeated target of natural selection in high‐elevation taxa (Aprelikova et al. 2004; Kaelin and Ratcliffe 2008). We found that a cytosine in an *Egln3* intron was hypermethylated in response to hypoxia in lowland mice and carried higher methylation levels in wild highland mice relative to wild lowland mice. We also report that this cytosine was located within a possible DMR as nearby cytosines also show the same direction of methylation change. Intronic DNA methylation is correlated with alternative splicing, with confirmation of such relying upon long‐read sequencing technologies to test for differential transcript usage. Further, concordant signatures of hypermethylation at *Egln3* in wild highland mice and lowland hypoxia‐treated mice may indicate an adaptive response. While we see no change in *Egln3* methylation in response to hypoxia in lab‐reared highland mice, higher methylation in wild highland mice (in comparison to wild lowland mice) may suggest that mice from these habitats abrogate their need to dynamically alter methylation at this site by co‐opting stable and increased methylation at *Egln3* to deal with chronic hypoxia. It is also possible that the derived missense mutation within the oxygen‐sensitive subunit of HIF2 (*Epas1*), which is regulated by *Egln3* and present in increased frequencies in highland mice (Ivy et al. 2022; Schweizer et al. 2019), overrides the need for this molecular plasticity since adaptive genetic variants within the same pathway can make the *Egln3* methylation response redundant. Molecular evidence pertaining to *Egln3* activity in both populations along with *Epas1* genotypic data can help resolve these possibilities. Nevertheless, our results suggest distinct reliance on molecular mechanisms enabling epigenomic plasticity among both populations.

We have shown that populations of deer mice that occupy the extreme ends of the elevation gradient exhibit geographic variation in methylation responses to hypoxia, with lowland mice generally exhibiting a more robust response than highlanders. These epigenetic changes are likely functionally relevant as they are concentrated at hypoxia‐relevant genes, specifically at *Egln3* in lowland mice, which is a regulator of *Epas1* and a gene that has experienced positive selection at high elevation in highlanders. Further functional research, though, is needed to validate the impact of these discovered methylation patterns. While we note that our sample sizes are relatively small, we are confident that our main conclusions are not driven by outlier effects. A greater magnitude of change in methylation between normoxia‐ and hypoxia‐exposed lowland mice (in comparison to lab‐born and reared highland mice) also supports the existence of a more robust epigenetic response to hypoxia in lowland mice, which may reflect its evolutionary origins as a response to transient or localised hypoxia in lowland environments.

## Author Contributions

Z.C., S.C.S. and B.M.H. designed the research study. Z.C. and S.C.S. collected the samples. B.M.H. generated the methylation data, and D.T. conducted the data analysis. All authors contributed towards the preparation and writing of the manuscript.

## Disclosure

Benefits generated: A research collaboration was developed with scientists who conducted either field‐ or laboratory‐based data; all collaborators are included as co‐authors, and the results of the research have been made available in public databases as described above.

## Ethics Statement

Sample collection and experimentation were carried out as per ethical codes established by Colorado Parks and Wildlife (CO CWP 17TR2168a), the United States Forest Service (Authorisation ID: CLC772) and the Institutional Animal Care and Use Committee (041–15).

## Conflicts of Interest

The authors declare no conflicts of interest.

## Supporting information


Data S1.

Data S2.

Data S3.

## Data Availability

We deposited raw FASTQ files on NCBI's public Short Read Archive under the accession PRJNA1035539. Processed DNA methylation data is available through Gene Expression Omnibus (GEO# GSE270202).
