# Supplementary material for: Geographic Variation in Epigenetic Responses to Hypoxia in Deer Mice ( Peromyscus maniculatus ) Distributed Along an Elevational Gradient
Source: Mol Ecol. 2025 Mar 28;34(9):e17752. doi: 10.1111/mec.17752 (PMC12010463; doi:10.1111/mec.17752)
Supplement: Supplementary file 3 — Data S3. [file MEC-34-e17752-s002.docx]

**Supplemental Information for:**

**Geographic variation in epigenetic responses to hypoxia in deer mice (Peromyscus maniculatus) distributed along an elevational gradient**

Dhriti Tandon, Shane Campbell-Staton, Zachary Cheviron, Bridgett M. vonHoldt

**Table of Contents:**

| **Materials and Methods** | Page 2 |
| --- | --- |
| **Supplemental Figures** | Page 3 |
| **Supplemental Tables** | Page 7 |
| **Custom Code** | Page 12 |

**Materials and Methods**

*Collection Permits and mouse care*

We collected wild mice under the approved permits from Colorado Parks and Wildlife (CO CWP 17TR2168a) and the United States Forest Service (Authorization ID: CLC772). We transferred all wild-caught individuals to the University of Montana (Missoula, Montana, USA; elevation 980 m above sea-level; standard PO2=19.1 kPa) to establish eight within-population mating pairs. We maintained the first-generation progeny of these eight breeding pairs at a constant temperature of 22°C, 12:12 light/dark schedule, and an ambient pressure until sexual maturity (typically by 60 days of age). We provided captive mice with food and water ad libitum. We housed 2-4 mice per cage. Hypoxic groups were returned to normoxia for a brief period (<1h), as we cleaned cages weekly during acclimations.

We also collected wild mice, which was permitted under Colorado Parks and Wildlife (CO CWP 17TR2168a) and the United States Forest Service (Authorization ID: CLC772). All animal handling protocols were approved by the University of Montana Institutional Animal Care and Use Committee.

*Bioinformatic processing*

We converted BS-Seeker “.CGmap” files for subsequent analyses with *methylKit v1.24.0* using custom code (Supplemental Information, page 12). We removed any significant outliers revealed by principal component analyses and we employed statistical approaches that do not assume equal variances among treatments. As a result, while our design may be underpowered to detect modest differences in methylation levels across treatments, systematic bias is unlikely.

**Figure S1.** Principal Component Analyses of RRBS data obtained from 28 *Peromyscus maniculatus* left ventricles tissues. **A)** Pie-chart showing the proportion of variance explained by all the principal components. Pie-colors correspond to the principal components they represent, as noted in the key on the right. The top 2 PCs explaining the highest amount of variance are PC1 (21.1%) and PC2 (4.46%), as indicated by the black font on the chart. **B)** CL_11937 (red) has the highest PC1 score (x axis) and stands out as an outlier with respect to all other samples (blue).


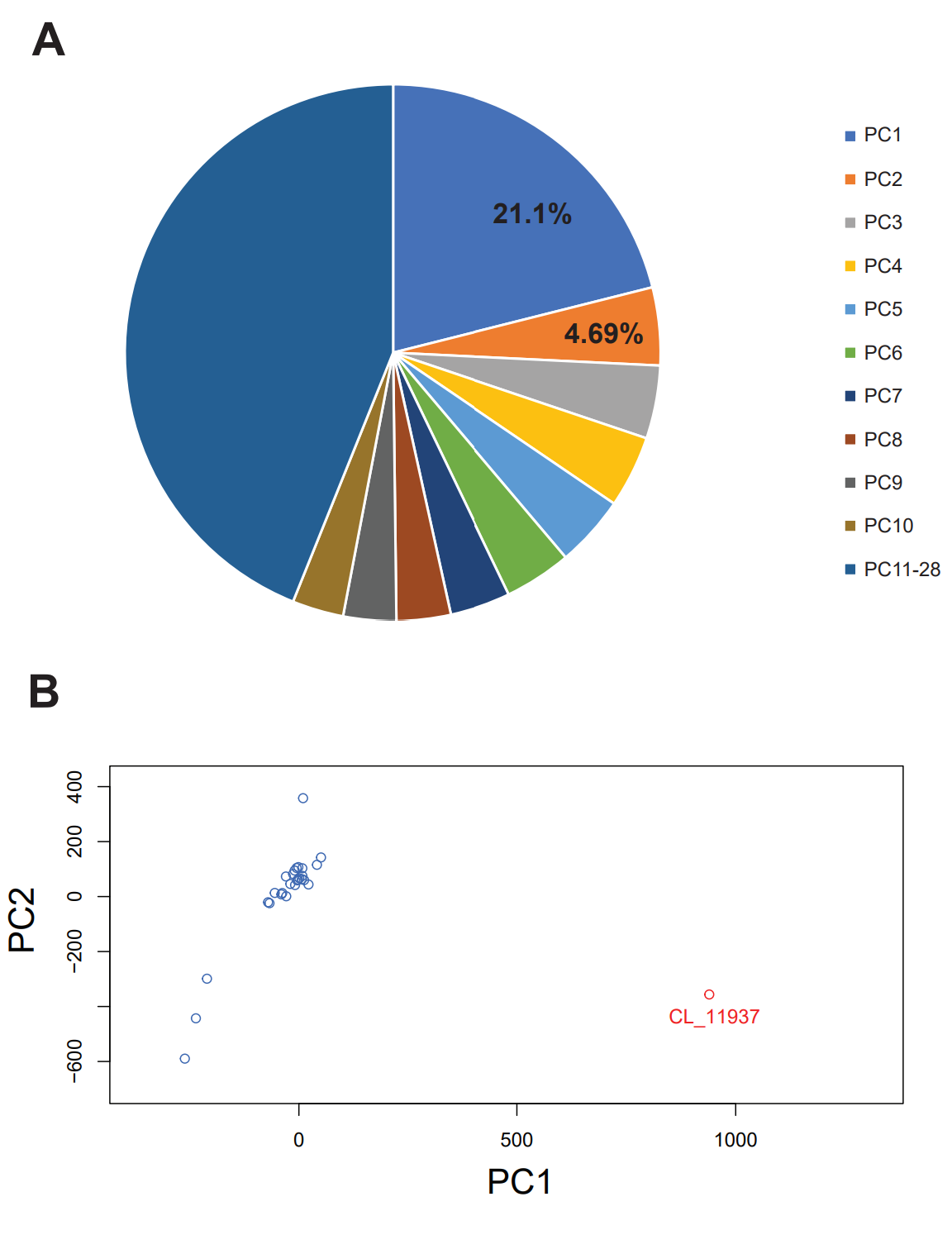


**Figure S2.** Methylation frequencies (y axis) in lab-reared highland mice by treatment (x axis). Bar chart depicting methylation frequency (y axis) among lab-reared highland mice at DMS located within **A)** *Egln3* *(chr14:16866983)* **B)** *Lrp1 (chr18:44654244)* and **C)** *Mapk13 (chr21:5927643).* Bar height corresponds to the mean value and error bar corresponds to standard errors. Bar height corresponds to the mean value and error bar corresponds to standard errors.

**
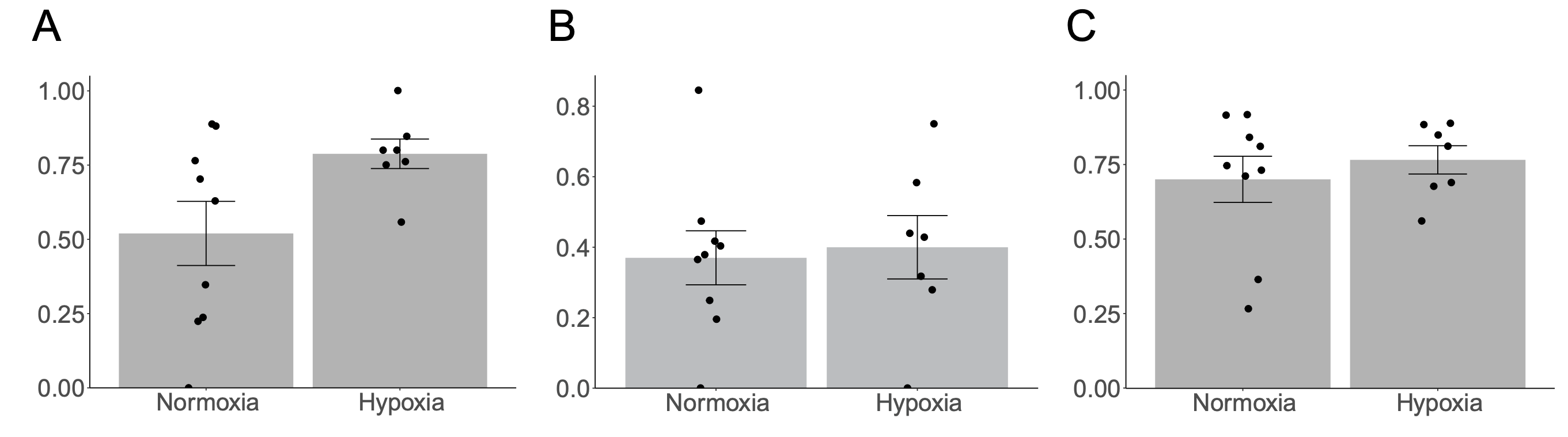
**

**Figure S3.** Percent of methylation differences (*methDiff*) at cytosines within ±1Kb flanking the shared differentially methylated site (indicated by *) for gene *Egln3* in **A)** lab-reared lowland and **B)** wild mice; gene *Lrp1* in **C)** lab-reared lowland and **D)** wild mice; and gene *Mapk13* in **E)** lab-reared lowland and **F)** wild mice. Red points denote cytosines with significant (*p*<0.05) methylation difference between normoxia versus hypoxia treated samples (for lab-reared lowland mice) and lowland versus highland (for wild mice). The horizontal dashed line indicates no methylation differences between two treatment groups. (Abbreviations: bp, base pair).

**
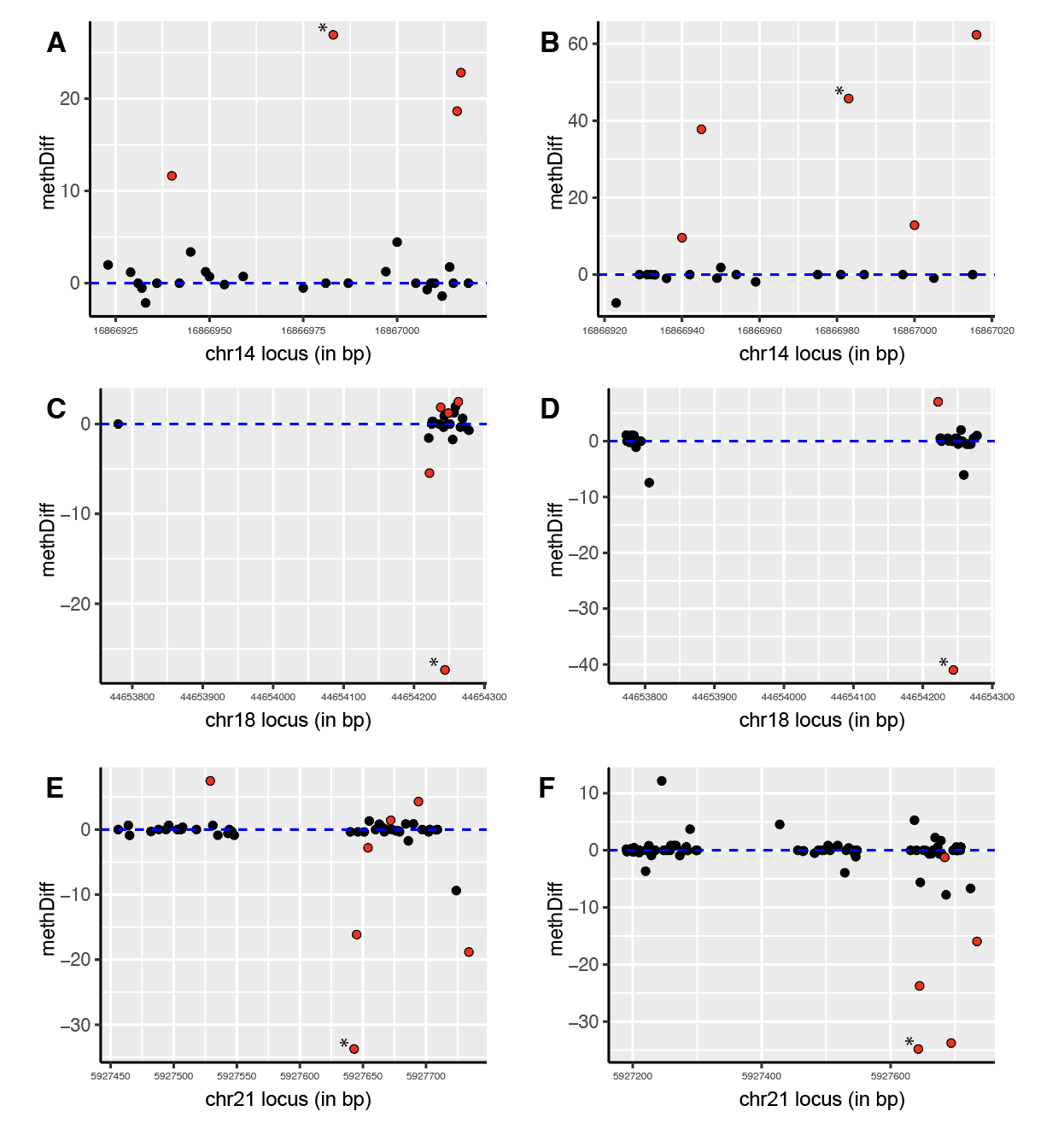
**

**Figure S4.** Percent of methylation differences (*meth_diff*) at cytosines within ±1Kb flanking the differentially methylated site (indicated by *) for gene *Rasgrp4* in **A)** lab-reared lowland, **B)** lab-reared highland, and **C)** wild mice. Red points denote cytosines with significant (*p*<0.05) methylation difference between normoxia versus hypoxia treated samples (for lab-reared mice) and lowland versus highland (for wild mice). The horizontal dashed line indicates no methylation differences between two treatment groups. (Abbreviations: bp, base pair).

**
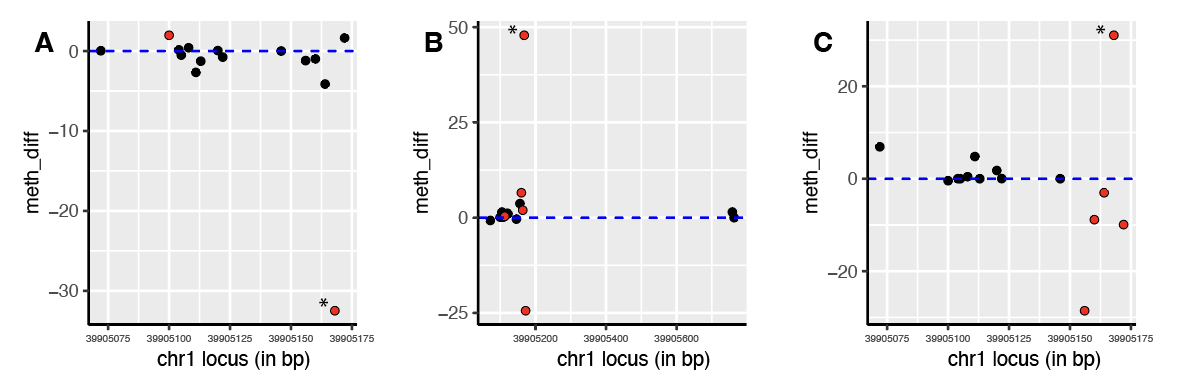
**

**Table S1.** Sample Metadata. (Abbreviations: F, female; M, male; NA, not applicable)

| Sample | Breeding-pair ID | Treatment* | Sex | Ecotype | Sequencing Pool |
| --- | --- | --- | --- | --- | --- |
| CL_7980 | F_47 | normoxia | F | Highland | Pool_5 |
| CL_7981 | F_43 | normoxia | M | Highland | Pool_3 |
| CL_7986 | F_36 | hypoxia | M | Highland | Pool_5 |
| CL_7995 | F_36 | hypoxia | F | Highland | Pool_2 |
| CL_7999 | F_43 | normoxia | M | Highland | Pool_2 |
| CL_8007 | F_36 | normoxia | F | Highland | Pool_5 |
| CL_8010 | F_36 | normoxia | F | Highland | Pool_1 |
| CL_11908 | F_113 | normoxia | F | Lowland | Pool_4 |
| CL_11909 | F_113 | normoxia | F | Lowland | Pool_6 |
| CL_11915 | F_132 | normoxia | M | Lowland | Pool_6 |
| CL_11916 | F_132 | normoxia | M | Lowland | Pool_4 |
| CL_11917 | F_132 | normoxia | M | Lowland | Pool_3 |
| CL_11918 | F_132 | hypoxia | M | Lowland | Pool_2 |
| CL_11919 | F_132 | normoxia | F | Lowland | Pool_6 |
| CL_11920 | F_132 | hypoxia | F | Lowland | Pool_6 |
| CL_11924 | F_132 | hypoxia | M | Lowland | Pool_3 |
| CL_11925 | F_132 | hypoxia | F | Lowland | Pool_3 |
| CL_11926 | F_132 | normoxia | M | Lowland | Pool_3 |
| CL_11934 | F_141 | normoxia | F | Highland | Pool_1 |
| CL_11935 | F_141 | hypoxia | F | Highland | Pool_5 |
| CL_11936 | F_141 | hypoxia | F | Highland | Pool_2 |
| CL_11937 | F_141 | normoxia | F | Highland | Pool_2 |
| CL_11943 | F_145 | normoxia | M | Highland | Pool_4 |
| CL_11945 | F_145 | hypoxia | F | Highland | Pool_4 |
| CL_11946 | F_146 | hypoxia | F | Highland | Pool_5 |
| CL_11948 | F_146 | hypoxia | F | Highland | Pool_3 |
| CL_11949 | F_146 | normoxia | F | Highland | Pool_1 |
| CL_11950 | F_146 | normoxia | F | Highland | Pool_2 |
| CL_7972 | NA | Wild-derived | M | Highland | Pool_4 |
| CL_7977 | NA | Wild-derived | M | Highland | Pool_2 |
| CL_7978 | NA | Wild-derived | M | Lowland | Pool_4 |
| CL_7982 | NA | Wild-derived | F | Highland | Pool_3 |
| CL_8001 | NA | Wild-derived | F | Lowland | Pool_3 |
| CL_8002 | NA | Wild-derived | M | Lowland | Pool_5 |
| CL_8006 | NA | Wild-derived | M | Highland | Pool_6 |
| CL_8008 | NA | Wild-derived | M | Highland | Pool_4 |
| CL_8011 | NA | Wild-derived | F | Lowland | Pool_1 |
| CL_8015 | NA | Wild-derived | F | Lowland | Pool_3 |
| CL_8018 | NA | Wild-derived | F | Highland | Pool_3 |

* indicates the experimental exposure of lab-reared mice to hypoxic or normoxic conditions.

**Table S2.** Reduced representation bisulfite sequencing library statistics after reads were aligned to the Pman_2.0 reference genome with mismatches <=4. (Abbreviations: N, number)

| Sample | Proportion of $\lambda$in bisulfite conversion | Percent methylation in motif: | | | N total bases | N of mapped that uniquely mapped | Percent of reads mapped |
| --- | --- | --- | --- | --- | --- | --- | --- |
|  |  | CG | CHG | CHH |  |  |  |
| CL_7980 | 0.996 | 20.2 | 2.6 | 1.6 | 6,184,226,035 | 35,668,269 | 58.1 |
| CL_7981 | 0.995 | 20.5 | 3.8 | 2.5 | 4,484,817,056 | 31,680,958 | 66.8 |
| CL_7986 | 0.996 | 18.0 | 3.9 | 2.7 | 3,071,107,113 | 20,162,171 | 62.7 |
| CL_7995 | 0.995 | 27.5 | 2.3 | 1.6 | 3,481,722,215 | 22,945,975 | 66.1 |
| CL_7999 | 0.995 | 29.1 | 2.2 | 1.7 | 4,970,520,670 | 32,563,609 | 65.9 |
| CL_8007 | 0.995 | 24.7 | 2.3 | 1.6 | 4,958,153,791 | 32,884,882 | 66.4 |
| CL_8010 | 0.995 | 16.5 | 4.1 | 2.6 | 6,272,005,192 | 43,792,196 | 65.4 |
| CL_11908 | 0.994 | 22.4 | 5.0 | 2.9 | 5,945,089,679 | 36,598,468 | 59.1 |
| CL_11909 | 0.995 | 25.8 | 1.9 | 1.4 | 4,969,611,599 | 31,934,809 | 64.3 |
| CL_11915 | 0.995 | 27.5 | 0.8 | 0.7 | 3,121,776,943 | 18,854,593 | 63.1 |
| CL_11916 | 0.995 | 25.1 | 3.0 | 1.8 | 4,682,094,535 | 26,698,854 | 56.9 |
| CL_11917 | 0.995 | 13.9 | 5.0 | 2.9 | 4,594,377,531 | 28,542,357 | 59.1 |
| CL_11918 | 0.995 | 27.3 | 1.8 | 1.4 | 2,902,688,899 | 19,119,735 | 65.9 |
| CL_11919 | 0.995 | 25.6 | 1.3 | 1.0 | 6,515,440,509 | 42,990,333 | 66.9 |
| CL_11920 | 0.995 | 21.6 | 1.8 | 1.3 | 3,828,165,752 | 25,838,726 | 68.1 |
| CL_11924 | 0.995 | 28.6 | 2.8 | 1.8 | 3,087,115,161 | 18,432,737 | 59.9 |
| CL_11925 | 0.995 | 30.3 | 1.5 | 1.0 | 4,240,293,411 | 24,315,099 | 58.6 |
| CL_11926 | 0.995 | 24.4 | 1.8 | 1.2 | 3,367,684,242 | 23,478,275 | 70.1 |
| CL_11934 | 0.994 | 30.6 | 2.7 | 1.9 | 3,108,722,866 | 19,838,320 | 62.9 |
| CL_11935 | 0.995 | 24.8 | 1.9 | 1.5 | 5,508,814,755 | 37,724,480 | 68.7 |
| CL_11936 | 0.995 | 23.3 | 5.0 | 3.2 | 2,911,912,955 | 20,492,574 | 65.3 |
| CL_11937 | 0.994 | 28.5 | 3.7 | 2.3 | 3,429,279,064 | 20,375,526 | 58.2 |
| CL_11943 | 0.995 | 29.5 | 1.9 | 1.4 | 4,937,493,841 | 31,460,543 | 64.9 |
| CL_11945 | 0.995 | 28.0 | 2.5 | 1.8 | 4,314,112,392 | 27,205,957 | 63.9 |
| CL_11946 | 0.995 | 32.9 | 1.0 | 0.8 | 3,354,666,783 | 21,678,764 | 67.6 |
| CL_11948 | 0.995 | 16.9 | 4.2 | 2.7 | 4,115,871,725 | 29,125,922 | 66.7 |
| CL_11949 | 0.995 | 26.4 | 2.8 | 1.9 | 3,558,473,481 | 21,266,395 | 59.9 |
| CL_11950 | 0.995 | 28.4 | 1.8 | 1.4 | 4,938,432,455 | 30,271,278 | 62.7 |
| CL_7972 | 0.995 | 25.1 | 2.3 | 1.6 | 2,035,251,648 | 20,199,476 | 65.9 |
| CL_7977 | 0.995 | 27.5 | 2.6 | 1.9 | 1,800,230,822 | 17,921,913 | 65.3 |
| CL_7978 | 0.950 | 28.4 | 1.3 | 1.0 | 1,928,032,688 | 18,510,582 | 66.7 |
| CL_7982 | 0.954 | 24.6 | 4.1 | 2.5 | 1,932,210,954 | 19,827,638 | 63.3 |
| CL_8001 | 0.957 | 19.6 | 1.7 | 1.1 | 2,452,199,736 | 24,067,784 | 63.3 |
| CL_8002 | 0.958 | 16.7 | 3.1 | 2.1 | 3,970,425,140 | 41,140,535 | 63.0 |
| CL_8006 | 0.956 | 28.8 | 2.3 | 1.5 | 2,742,680,092 | 27,315,802 | 61.6 |
| CL_8008 | 0.959 | 19.7 | 3.9 | 2.4 | 2,192,113,400 | 22,318,601 | 59.8 |
| CL_8011 | 0.955 | 20.0 | 2.3 | 1.9 | 2,933,946,413 | 28,892,105 | 61.0 |
| CL_8015 | 0.950 | 27.0 | 1.0 | 0.9 | 1,541,511,701 | 14,854,071 | 67.1 |
| CL_8018 | 0.953 | 25.8 | 2.0 | 1.4 | 2,529,387,942 | 24,714,064 | 63.3 |

**Table S3.** Common differentially methylated sites among lowland and highland mice in response to hypoxia in the Pman_2.0 reference genome.

|  | Directional change in methylation in: | |  | |  | |
| --- | --- | --- | --- | --- | --- | --- |
| Chr.position | Lowland | Highland | | Ensembl ID | | Gene Symbol |
| chr1.17666383 | 32.61 | -35.54 | | ENSPEMG00000016882 | | *Dact3* |
| chr1.39905168 | -32.49 | 47.88 | | ENSPEMG00000018935 | | *Fam98c* |
| chr3.60374638 | -30.66 | 45.98 | | Intergenic | | *Intergenic* |
| chr5.34277582 | -25.58 | -26.13 | | ENSPEMG00000018749 | | *Slc12a4* |
| chr8.44633624 | -25.33 | 25.41 | | ENSPEMG00000025274 | | *Natd1* |
| chr8.53797109 | 26.80 | -31.68 | | ENSPEMG00000019217 | | *Tnfsf13** |
| chr14.78976483 | 30.47 | 34.71 | | Intergenic | | *Intergenic* |

*hypoxia relevant gene

**Custom Code**

Code used to convert “.CGmap” output files (produced by *bs_seeker2-call-methylation.py* script in *BS-Seeker2 v2.0.10*) to *methylKit v1.24.0*  input-

awk <*SampleID*_pma2_methyl.CGmap ' { print $1"."$3, $1, $3, "F", $8, $6*100, (1-$6)*100 } ' > *SampleID*_pma2_for_methylKit.txt
